# Axon outgrowth and neuronal differentiation defects after a-SMN and FL-SMN silencing in primary hippocampal cultures

**DOI:** 10.1371/journal.pone.0199105

**Published:** 2018-06-14

**Authors:** Daniela Pletto, Silvia Capra, Adele Finardi, Francesca Colciaghi, Paola Nobili, Giorgio Stefano Battaglia, Denise Locatelli, Cinzia Cagnoli

**Affiliations:** Molecular Neuroanatomy and Pathogenesis Unit, Neurology VII—Clinical and Experimental Epileptology Unit, Foundation IRCCS Neurological Institute “C. Besta”, Milano, Italy; University of Louisville, UNITED STATES

## Abstract

Spinal Muscular Atrophy (SMA) is a severe autosomal recessive disease characterized by selective motor neuron degeneration, caused by disruptions of the Survival of Motor Neuron 1 (*Smn1*) gene. The main product of SMN1 is the full-length SMN protein (FL-SMN), that plays an established role in mRNA splicing. FL-SMN is also involved in neurite outgrowth and axonal transport. A shorter SMN isoform, axonal-SMN or a-SMN, displays a more specific axonal localization and has remarkable axonogenic properties in NSC-34. Introduction of known SMA mutations into the a-SMN transcript leads to impairment of axon growth and morphological defects similar to those observed in SMA patients and animal models. Although there is increasing evidence for the relevance of SMN axonal functions in SMA pathogenesis, the specific contributions of FL-SMN and a-SMN are not known yet. This work aimed to analyze the differential roles of FL-SMN and a-SMN in axon outgrowth and in neuronal homeostasis during differentiation of neurons into a mature phenotype. We employed primary cultures of hippocampal neurons as a well-defined model of polarization and differentiation. By analyzing subcellular localization, we showed that a-SMN is preferentially localized in the growing axonal compartment. By specifically silencing FL-SMN or a-SMN proteins, we demonstrated that both proteins play a role in axon growth, as their selective down-regulation reduces axon length without affecting dendritic arborization. a-SMN silencing, and in minor extent FL-SMN silencing, resulted in the growth of multi-neuritic neurons, impaired in the differentiation process of selecting a single axon out of multiple neurites. In these neurons, neurites often display mixed axonal and dendritic markers and abnormal distribution of the axonal initial segment protein Ankirin G, suggesting loss of neuronal polarity. Our results indicate that a-SMN and FL-SMN are needed for neuronal polarization and organization of axonal and dendritic compartments, processes that are fundamental for neuronal function and survival.

## Introduction

Spinal Muscular Atrophy or SMA is a severe autosomal recessive disease and the leading genetic cause of infant mortality. SMA has an incidence of 1 in 6.000–10.000 neonates and an estimated carrier frequency of 1 in 35 [1–3]. From a clinical standpoint, SMA is primarily characterized by motor neuron degeneration leading to progressive amyotrophic paralysis, respiratory failure, and, in more severe cases, death. The death of motor neurons, whose mechanism is currently unexplained, starts during early post-natal stage and continues until the later stages of development [4–8]. However, neuron loss is not limited to motor neurons [9–13]. Indeed, classical neuropathologic studies and recent data from both affected patients and murine SMA models already demonstrated that other cell types such as pyramidal neurons and glia cells are affected in SMA [14–21] as well as abnormalities in non-neuronal systems [22–25, 18, 13, 26, 27], suggesting multiple pathogenetic pathways leading to the SMA phenotype.

In humans, the SMA disease gene *SMN1* (survival motor neuron 1) [28] produces the functional full-length SMN protein (FL-SMN). The duplicated, almost identical *SMN2* copy gene governs disease severity by producing an exon 7 truncated SMN form (Δ7-SMN) and only low amounts of FL-SMN [29–31]. An additional, truncated isoform of SMN, translated from an SMN1-derived mRNA retaining intron 3 and termed axonal SMN (a-SMN), is mainly expressed in axons in vivo and in vitro [32].

The FL-SMN protein is ubiquitously expressed and mainly localized in the cytoplasm and nuclear gems (gemini of Cajal bodies) in every cell type. FL-SMN mainly functions as an assembly factor for small nuclear ribonucleoprotein particles (snRNPs) or small nucleolar RNPs (snoRNPs) involved in pre-mRNA splicing [33–36]. FL-SMN is also localized in axons [37], associated with ribonucleoprotein granules and proteins involved in actin dynamics, mRNA transport, local translation and axon outgrowth [38–44], thus suggesting that SMN loss of function in axons might contribute to the pathophysiology of SMA.

As far as a-SMN is concerned, its specific contribution to the pathogenesis of SMA still needs to be clarified. Previous work has shown that a-SMN plays a role in axon outgrowth [32], through the C-terminus encoded by intron 3 [45]. On the other hand, the identical sequence in the N-terminal part between FL-SMN and a-SMN has not allowed to determine the true role of these two SMN isoforms in axonal growth/maintenance.

To verify the differential contribution of the two SMN protein isoforms to axon growth/neuronal differentiation, we used in the present paper a long-established in vitro setting, i.e., sandwich co-cultures of primary hippocampal neurons and glia. Embryonic hippocampal neurons in culture display well-defined and precise morphological steps of polarization and differentiation [46]. By applying specific small interference RNAs (siRNAs) efficiently down-regulating either FL-SMN or a-SMN proteins, we here analyzed the differential roles of FL-SMN and a-SMN in axon outgrowth and in neuronal homeostasis during differentiation of hippocampal neurons into a mature phenotype.

## Materials and methods

### Ethic statement

All the procedures involving animals were performed in accordance with national (DL 116/1992 and DL 26/2014), and European Community Council guidelines (EEC Council Directive 86/609/EEC, Guide for the Care and Use of Laboratory Animals, and Directive 2010/63/EU, Legislation for the protection of animals used for scientific purposes). The experimental protocol was approved by the Ethics Committee of the “C. Besta” Neurological Institute and by the Italian Ministry of Health (protocol number: BR2/2014). Particular care was taken to minimize the number of animals, their discomfort and pain.

### Cell cultures

In all experiments with primary cultures, sandwich co-cultured glial cells and hippocampal neurons were used [47]. Primary cultures of astrocyte as feeder layer were prepared from the cerebral cortex of 1-day-old rat pups. Pups were euthanized by decapitation after anesthesia with isoflurane. Dissected hemispheres were cut in thin sections, incubated at 37°C in Hank’s balanced salt solution (HBSS; Life Technologies, Carlsbad, CA, USA), containing 10 mM HEPES (Life technologies) with 0.25% trypsin (Sigma-Aldrich, St. Louis, MO, USA) and 0.05 mg/ml Dnase I (Sigma-Aldrich n° DN25), and passed through a cell strainer with 70-μm mesh (BD Biosciences, Franklin Lakes, NJ, USA). The suspension was seeded at the density of 2 x 10^2^ cells/cm^2^ in 75 cm^2^ culture flask (Nunc®, Penfield, New York, USA) in Minimal Essential Medium (MEM; Gibco®, Grand Island, NY, USA) with Earle’s salts and L-glutamine supplemented with 10% heat-inactivated fetal bovine serum (FBS; Thermo Scientific, Walyham, MA, USA), 0.6% glucose (Sigma-Aldrich) and 1% penicillin/streptomycin. Seven to ten days after dissection, cultures were transferred into 60 mm dishes (Nunc®) in Minimal Essential Medium with Earle’s salts and L-glutamine and supplemented with 0.6% glucose and 10% heat-inactivated horse serum (HS; Gibco®).

Primary hippocampal neurons were prepared from embryonic (E18) Sprague-Dawley rats (Charles River, Calco, Italy), as described previously [47]. Pregnant mother was euthanized by decapitation after anesthesia with isoflurane. Fetuses were dissected out and sacrificed by decapitation. Hippocampal regions were quickly exposed and dissected out. Neurons were acutely dissociated for 15 min at 37°C with 0.25% trypsin (Sigma-Aldrich) and triturated through a fire-polished Pasteur pipette. Cells were then plated on glass coverslips (Gerhard Menzel, Glasbearbeitungswerk GmbH & Co. KG, Braunschweig, Germany) pre-coated with 1 mg/ml poly-L-lysine solution (Sigma-Aldrich) at a density of 100–200 cells per mm^2^ for 4 hours in MEM/HS Medium. Hippocampal neurons were then flipped over astrocyte cultures in 6 cm dish and maintained in MEM medium containing the N2 supplement (Gibco®), grown in a humidified 95% air and 5% CO_2_ atmosphere at 37°C. Experiments were performed in cultures maintained up to 8 days in vitro (8 DIV).

NSC34 (hybrid mouse neuroblastoma/motor neuron cell line) [48] cells were routinely grown at 37°C in a humidified atmosphere (5% CO_2_−95% air) in 25 cm^2^ culture flasks (Corning, Cambridge, MA, USA) in Dulbecco’s modified Eagle’s medium (DMEM, GLUTAMAX™; Gibco®) supplemented with 5% heat-inactivated FBS and antibiotics (penicillin G K-salt, 100 UI/mL and streptomycin sulphate, 100 μg/mL). The culture medium was replaced every 2–3 days. Every week cells were detached from the plates by mechanical dissociation in culture medium, and then replated at a density of 5 X 10^4^ cells/ flask.

### Plasmid generation and cell transfection

PCR-amplified and gel-purified a-SMN (encompassing exon 1 to intron 3) or FL-SMN fragments were cloned in frame in the pcDNA4/HisMaxTOPO expression vector (Life Technologies)[32]. All obtained clones were fully sequenced. For co-transfection experiments in NSC34 cells, plasmid DNAs were transfected with (Dicer-Substrate) 27-mer siRNAs (DsiRNA; IDT, Coralville, IA, USA) using Lipofectamine® 2.000 (Life Technologies) in 2:1 reagent/DNA ratio under previously optimized conditions and a final 100 pmol concentration of siRNAs. For co-transfection experiments in primary hippocampal neurons DsiRNAs were transfected with the pCAG-Venus–EGFP (kind gift from F. Calderon de Anda) plasmid encoding a green fluorescent protein fully labelling cell bodies and neurites for length measurements (see below).

### Cell staining and immunofluorescence

Neurons were fixed in 1% or 4% paraformaldehyde in phosphate-buffered saline (PBS) with 1.44 M sucrose, 2 mM EGTA and 3 mM MgCl_2_ at 37°C. After quenching with 50 mM ammonium chloride (NH_4_Cl) neurons were permeabilized with 0.1% Triton X-100 in PBS, and non-specific binding was blocked in 2% normal goat serum (NGS; Vector laboratories Inc., Burlingame, CA, USA), 2% bovine serum albumin (BSA; Sigma-Aldrich) and 0.2% fish gelatine in PBS. Neurons were then incubated with primary antibodies diluted in 10% blocking solution at 4°C overnight. The secondary antibodies Alexa Fluor® 546, 488, 647, or pacific blue conjugated goat anti-mouse, anti-rabbit, anti-chicken (Life Technologies) were incubated for 1 h at room temperature. To quantify a-SMN staining intensity, primary neurons were co-labelled with 5-(4,6-dichlorotriazinyl)aminofluorescein (5-DTAF 10 μg/ml; Life technologies). Cells were repeatedly rinsed, coverslipped with Fluoroshield™ (Sigma-Aldrich) and examined on a TCS SP8 confocal microscope (Leica Microsystems, Wetzlar, Germany). Primary antibodies were: i) rabbit polyclonal rat-specific (#553) anti-a-SMN antibodies directed against the C-terminal region of the a-SMN sequence (diluted 1:1.000; NeoMPS, Strasbourg, France); ii) mouse monoclonal anti-SMN (diluted 1:1.000: clone 8, BD Biosciences); iii) rabbit polyclonal anti-SMN (H-195) (diluted 1:1000, Santa Cruz Biotechnology, Santa Cruz, CA, USA) iv) mouse monoclonal (diluted 1:1.000; Merck-Millipore, Billerica, MA, USA) or rabbit polyclonal (diluted 1:1.000; Covance, Princeton, NJ, USA) anti-III-β-tubulin; v) mouse monoclonal anti-phosphorylated neurofilaments SMI31 (diluted 1:1.000, Covance); vi) mouse monoclonal anti-Tau-1 (diluted 1:500; Merck-Millipore); vii) rabbit polyclonal (diluted 1:1000; Cell Signalling, Danvers, MA, USA) or mouse monoclonal (diluted 1:100; NeoMarkers, Fremont, CA, USA) anti-MAP2; viii) mouse monoclonal anti-ankyrin G (diluted 1:15; SantaCruz Biotechnology).

### RNA interference

Two different gene-specific siRNA oligonucleotides targeting either FL-SMN or a-SMN and an additional control siRNA were obtained from IDT (Dicer-substrate 27-mer Duplex RNAs D-siRNA). The rat FL-SMN cDNA sequence (GenBank accession n° NM_022509.1) and a-SMN cDNA sequence (GenBank accession n°AY876898) were used for selecting the target regions to be silenced using the IDT RNAi Design tool. Criteria for sequence selection were: length of 21 nucleotides, 30–50% GC content, no stretches of > 4Ts or As. Sequence analysis using the BLAST algorithm (NCBI) gave no significant match to other targets. We selected two specific siRNAs targeting either the exon 3/exon 4 junction of rat FL-SMN (IDT, Reference n° 62956205; sequence of sense strand from nucleotide position 491 to 517: 5’-ACAGAACACTCAGGAGAATGAAAGCCA-3’) or the rat a-SMN intron 3 (IDT, Reference n° 62956208; sequence of sense strand from nucleotide position 530 to 556 5’-TCCTGAAAGTGAGGAGAAACAGCGTTG-3’). As negative control a non-targeting sequence in the human, mouse, or rat transcriptomes (IDT, Reference n° 64438470, IDT) was used.

For silencing hippocampal neurons, freshly isolated neurons were co-electroporated in suspension with 1 μg Venus plasmid DNA plus 0.5 μg siRNAs using the AMAXA™ Nucleofector™ System (Lonza Inc., Allendale, NJ, USA). Post nucleofection, neurons were immediately plated on coated glass coverslips in MEM/HS medium in a humidified 95% air and 5% CO_2_ atmosphere at 37°C. After 4 hours neurons were flipped over astrocyte cultures in 6 cm dish and maintained in Neuronal Maintenance Medium. After 3 DIV (at developmental stage 3) or 8 DIV (stage 4, to allow ankyrinG immunostaining in the axon initial segment) [49], neurons were fixed with 4% or 1% PFA for IF or confocal analysis.

To quantify silencing efficiency at the protein level western blot analysis of NSC34 cell lysates was performed. NSC34 cells were plated on 25 cm^2^ culture flask (Corning), and transfected at 80–90% confluency with 8 μg of either tagged FL-SMN or tagged a-SMN cDNAs plus 3 μg of siRNA using 3 μg/ml Lipofectamine® 2.000 (Life Technologies n° 11668) in serum-free medium. After transfection, cultures were maintained for 72 hours in DMEM GLUTAMAX™ medium and then collected as described below.

### Western blot analysis

Co-transfected NSC34 cells were lysed in buffer containing 0.1 M Sodium phosphate (pH 7.4), 0.2% Triton X-100, 0.1 mM EDTA, 0.2 mM phenylmethanesulfonyl fluoride (PMSF), 1 mg/mL aprotinin and 1 mg/mL leupeptin by three repeated freezing and thawing cycles. The resulting cell lysates were centrifuged at 13.000 g. Proteins were separated by sodium dodecyl sulphate–polyacrylamide gel electrophoresis (12% acrylamide) and electro-blotted on nitrocellulose paper for 1 h at 180 mA. The nitrocellulose was blocked overnight with 5% no-fat milk in Tris-buffered saline (TBS). The primary antibodies anti-SMN antibody (1:15.000; BD Bioscience), directed against the N-terminus of the FL-SMN sequence and of the a-SMN sequence, mouse monoclonal anti-tag Xpress (1:1.000; Life technologies n° R910-25) and α-tubulin (1:5.000; Sigma-Aldrich) were diluted in 5% no-fat milk in TBS and incubated with the nitrocellulose for 1.5 hours. The membranes were rinsed in TBS–Tween 20, and incubated respectively with IRDye® 800- and IRDye® 680- goat anti-mouse secondary antibodies (1:15.000; LI-COR, Lincoln, NE, USA), in 5% no-fat milk in TBS for 45 min. After washes in TBS-Tween 20, the Odyssey Infrared Imaging System (LI-COR) was used to measure protein concentration. Scanning parameters (non-saturated signals, resolution of 84 or 169 mm for high/medium quality) were set according to the manufacturer’s instructions. Outlines were drawn around the bands and the integrated intensity was calculated after subtracting background. The amount of SMN proteins was normalized versus α-tubulin signals and compared among groups.

To test the specificity of #553 anti-rat a-SMN antibody (NeoMPS, Strasbourg, France), E14 rat embryo whole brain, or E16 rat embryo brain or hippocampus were lysed with a tissue potter in buffer containing 20mM HEPES (pH 4,4), 1mM 1,4-dithiothreitol (DTT), 1mM EDTA, 0,1mM PMSF, Complete protease inhibitor (diluted 1:25; Roche Applied Science, Mannheim, Germany). The lysates were centrifuged at 10,000xg and surnatant proteins were subjected to western blot analysis as described above. The nitrocellulose was incubated with #553 antibody (1:1000) overnight at 4°C. After incubation with IRDye® 800 goat anti-rabbit secondary antibody, the same nitrocellulose was subsequently probed with anti-SMN antibody (1:15,000; BD Bioscience) and mouse anti-actin antibody (1:10,000; Chemicon International, Temecula, CA), followed by the IRDye® 680 goat anti-mouse secondary antibody, and images acquired with Odyssey Infrared Imaging System.

### Morphometric and statistical analysis

To quantify the a-SMN fluorescence signal in neuritic processes during neuronal differentiation, at least 30 neurons at each stage of differentiation (2, 2+ and 3) were analyzed from three different experiments. 20x magnification images were acquired. To make sure that the signal was not saturated in neurites of the cells imaged for analysis, exposure settings for acquisition were selected using color LUT (Look up table) tool of Leica LAS suite software for confocal imaging, and were held constant for all acquisitions for each experiment. Lines were traced along the entire extension of axons (identified as the longest neurite) and dendrites, average background fluorescence intensity was determined with ImageJ (W. Rasband, National Institutes of Health, Bethesda, MD) and subtracted from the axonal and dendritic intensity values [50]. The intensities of all neuritic segments considered were then averaged and divided for the corresponding mean grey value of the green fluorescent dye (5- DTAF). In stage 3 neurons, axons were identified as the longest neurite, which was also labeled by anti-tau antibody.

For representative images of neurons at stage 2, stage 2+ and stage 3 at higher magnification (40x or 63x), pseudocolor images of fluorescence intesity ratio heat maps were obtained calculating the a-SMN/DTAF ratio with the “Ratio Plus” ImageJ plugin after background subtraction. The colder blue color (0) was assigned to a ratio value of 0 and the warmer white color (255) to the higher ratio value (2) [51, 52].

To evaluate morphology effects after silencing, the identification of axons and dendrites in 3DIV neurons was based on morphological parameters, as described [53]. Three types of Venus^+^ and III-β-tubulin^+^ neurons were identified for analysis based on morphological criteria: i) symmetric stage 2 neurons, when showing equally short minor neurites; ii) stage 3 neurons, when they formed a single prevailing axon, identified by its length as either >80 μm, or at least twice longer than the second longest neurite; iii) multi-neuritic neurons, when they extended two or more neurites longer than 40 μm (i.e., longer than normal minor neurites) without showing a prevailing axon. For neurites length measurement on 3DIV hippocampal cultures transfected with control or specific a-SMN or FL-SMN siRNA, individual Venus-expressing neurons in stage 3 (n = 50 cells from 3 different coverslips per experiment, n = 3 experiments per group) were randomly chosen and photographed with 40x or 63x magnification using TCS SP8 confocal microscope. All axonal and dendritic branches were traced, their lengths measured by manual outlining with the analysis tool of TCS SP8 confocal microscope and their numbers manually counted. We measured axons from the cell body to the growth cone (axon length), plus all axonal branches longer than 20 μm (total axon length).

All Venus^+^ multi-neuritic neurons, were also analyzed for their expression of axonal markers Tau-1 or SMI31.

### Statistical evaluation

The significance of the difference in a-SMN fluorescence intensity in primary neurites and primary axons *vs*. minor neurites and the efficiency of silencing were evaluated by unpaired Student’s t-test. Differences in morphometric measures and in percentage of “multi-neuritic” cells after silencing with control, FL-SMN or a-SMN siRNA were performed by means of one-way ANOVA followed by Tukey HSD as post-hoc comparison test, using a free web utility (http://vassarstats.net/). All the other statistical analyses were performed by chi-square test. All data were expressed as mean ± SEM of three independent experiments, and difference considered significant when p<0.05.

## Results

### a-SMN subcellular localization during neuronal differentiation

**Fig 1:**
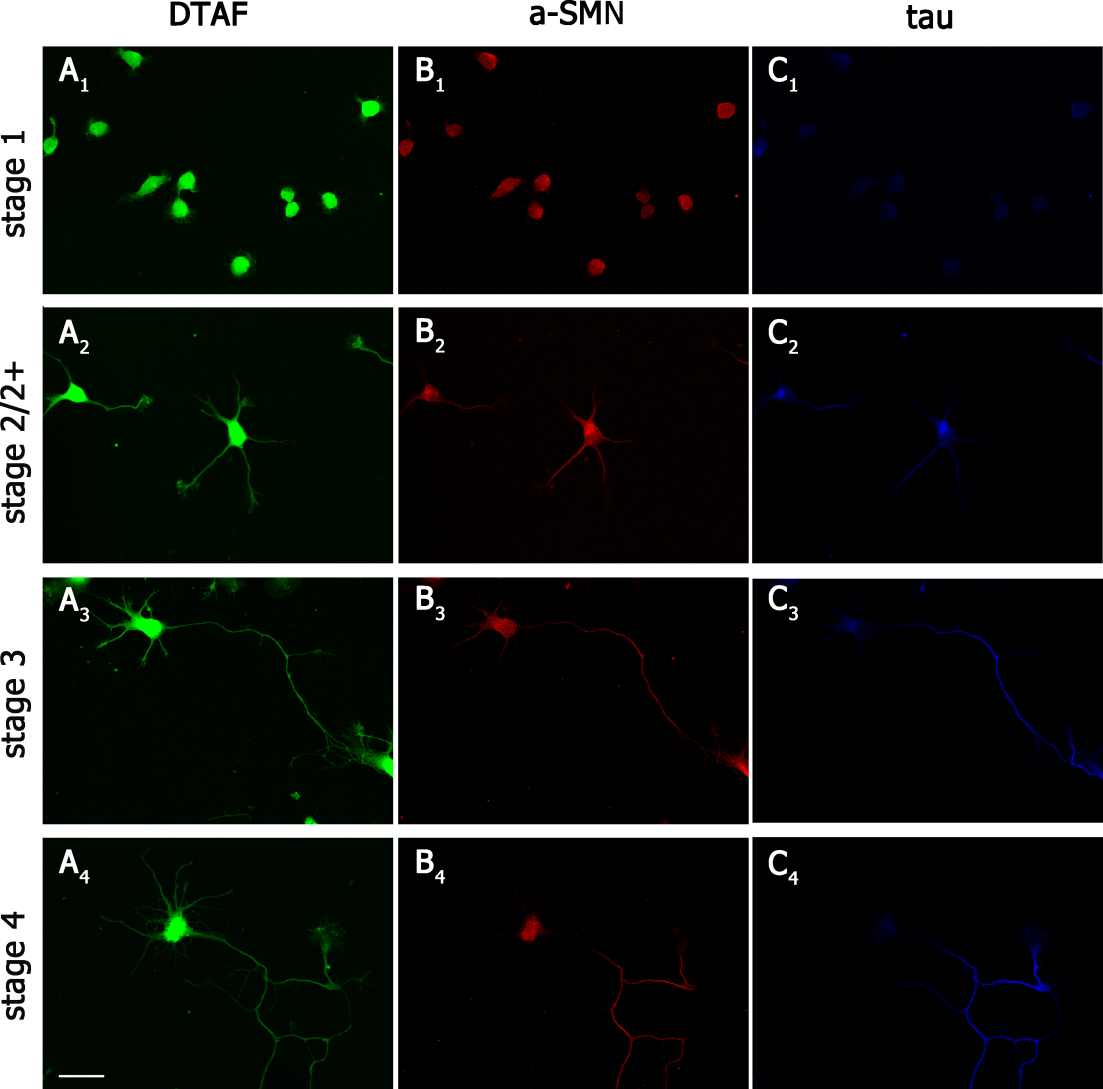
a-SMN subcellular localization during neuronal differentiation. Confocal images of primary hippocampal neurons from embryonic rats co-labeled with DTAF; (A_1_-A_4_, green), rat-specific anti-a-SMN antibody #553 (B_1_-B_4,_ red) and axonal marker anti-tau antibody (C_1_-C_4,_ blue). Note the early a-SMN staining within cell bodies in stage 1 (B_1_) and newly formed primary neurites in stage 2/2^+^ (B_2_), and the more selective staining of the forming primary axon in stages 2^+^-4 (B_3-4_), with a distribution similar to tau axonal staining (C_2_-C_4_). Scale bar: 25 μm.

We first verified the subcellular localization of a-SMN during different developmental stages of cultured primary hippocampal neurons. Dissociated hippocampal neurons derived from rat embryos become polarized *in vitro* following a reproducible and well characterized program [46]. Shortly after plating, hippocampal neurons extend a lamella around the cell body (stage1), which then coalesces to form several minor neurites (stage 2). Within 12–36 hours, one of the neurites starts to elongate very rapidly and overgrows the other neurites (stage 3). The fast growing neurite will become the future axon whereas the other neurites will constitute the dendrites at later stages. To allow proper visualization of fine cellular details, cultured neurons were prepared from E18 Sprague-Dawley rats and co-labeled with 5-DTAF (Fig 1A_1_–A_4_) and #553 rat-specific anti-a-SMN antibodies (Fig 1B_1_–B_4_). # 553 antibody specficity was verified by western blot analysis on rat E14 and E16 embryo cortex and hippocampus lysates, where # 553 antibody evidenced a specific immunoreactive band of approximately 29 KDa, distinct from the band corresponding to FL-SMN (~37kDa) recognized by the BD Bioscience anti-SMN antibody (S1 Fig). Confocal immunofluorescence (IF) analysis of hippocampal neurons revealed that a-SMN was localized in the cell body of stage 1 cells and excluded from lamellae (Fig 1B_1_).

**Fig 2:**
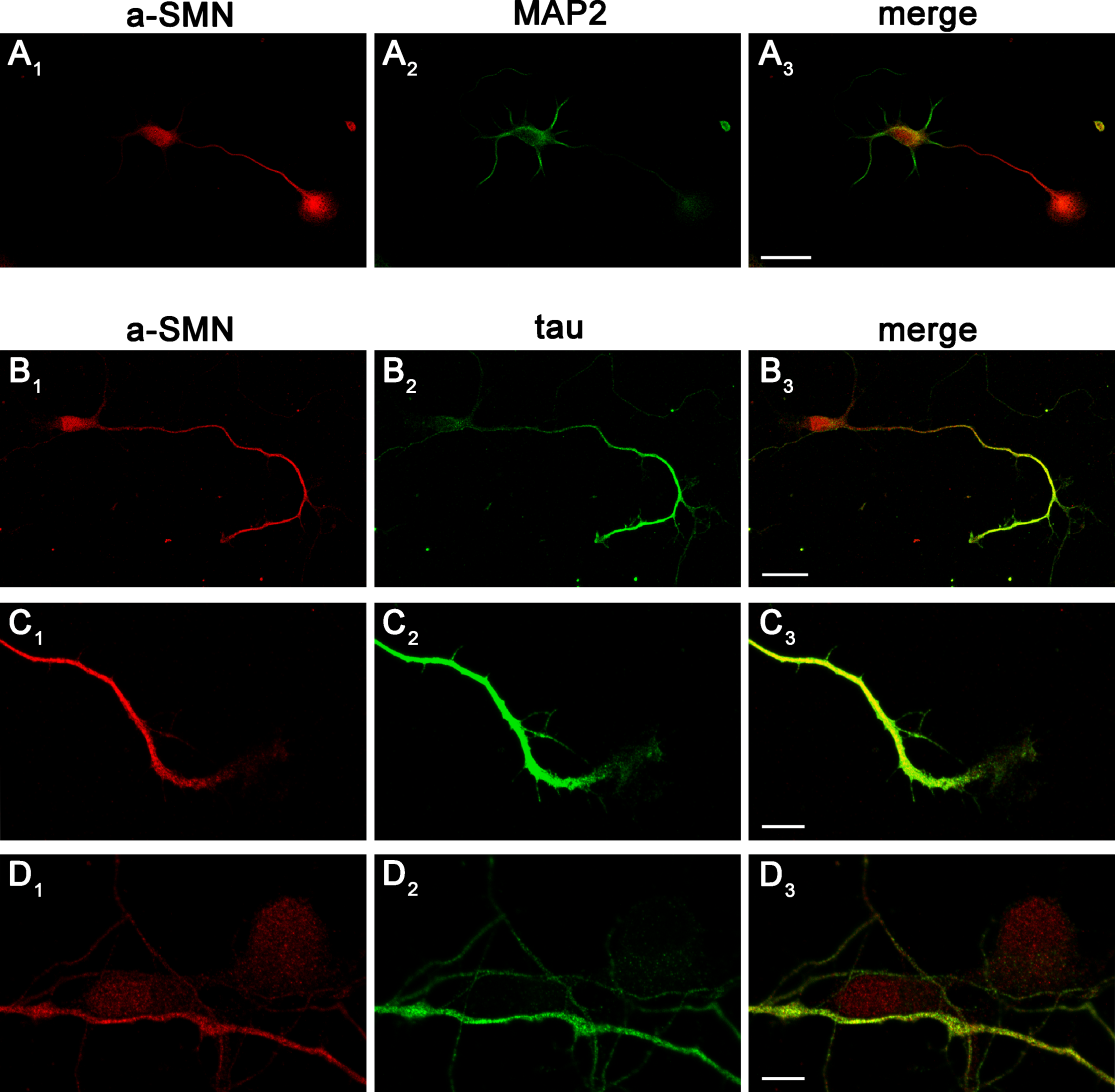
a-SMN subcellular localization in stage 4 neurons. Confocal images of primary hippocampal neurons from E18 embryonic rats co-labeled with #553 anti-rat-a-SMN (A_1_–D_1_, red) and anti-MAP2 antibody (A_2_, green) or anti-tau antibody (B_2_-D_2,_ green). Merged images are shown in column 3 (A_3_-D_3_). Note how a-SMN fluorescence shows a preferential localization in cell soma and axon, similar to tau (B_1_-B_3_) while it is less intense in the dendritic compartment, highlighted by dendritic marker MAP2 (A_1_-A_3_). Higher magnification images show how a-SMN staining is less intense at the growth cone tip (C_1_-C_3_). In the cell soma a-SMN fluorescence presents a punctuate pattern (D_1_-D_3_) with localization both cytoplasmic and nuclear, while tau staining is very low in the cytoplasm and nearly absent from the nucleus (D_2_). Scale bars: 25μm in A and B; 7,5μm in C and D.

As neuronal polarization progressed from stage 2/2^+^ (one neurite slightly longer) to stage 3 and stage 4 cells a-SMN became enriched in the longest neurite, the future axon, showing a pattern of localization similar to the axonal marker tau (Fig 1B_2_–C_4_). In particular, in neurons at stage 4, a-SMN was preferentially localized in the cell soma and axon (Fig 2B_1_–B_3_) compared to the dendritic compartment (Fig 2A_1_–A_3_). a-SMN fluorescence was uniformly distributed along the axon, except at the growth cone tips, which were usually less intensely stained (Fig 2C_1_–C_3_). In the cell body, a-SMN immunofluorescence showed a punctuate pattern present in both cytoplasm and nucleus (Fig 2D_1_–D_3_).

**Fig 3:**
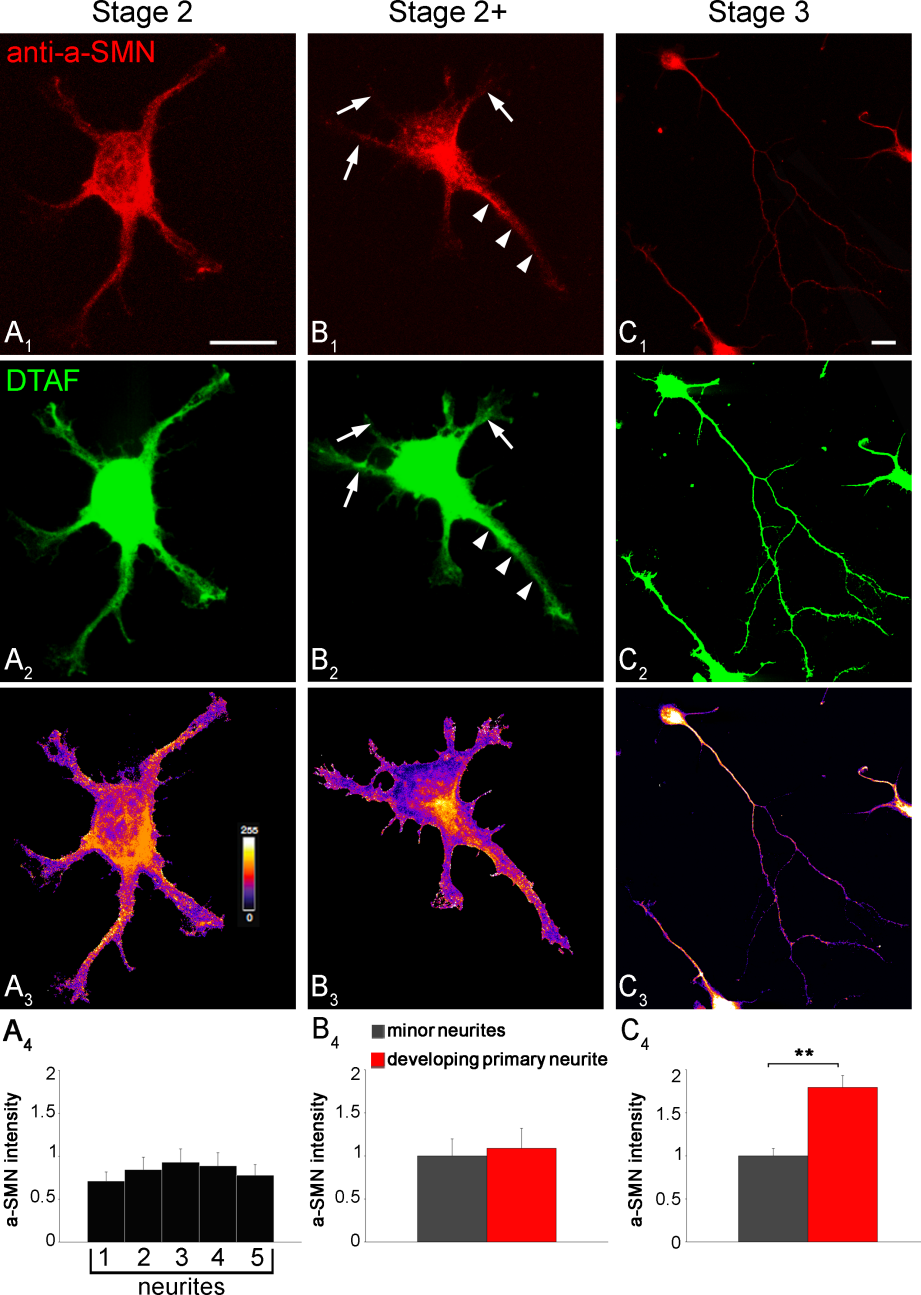
Quantification of a-SMN neuritic labeling during early neuronal differentiation. Stage 2–3 rat primary hippocampal neurons were co-labeled with the rat-specific anti-a-SMN antibody #553 (A_1_-C_1_, red) and the pan-cellular marker 5-DTAF (A_2_-C_2_, green). (A_3_-C_3_) Pseudocolor images showing heat maps of fluorescence intensity ratio between a-SMN and DTAF, with warm colors denoting higher signal (0–255). (A_4_-C_4_) Intensity quantification expressed as a-SMN/DTAF fluorescence ratio. The a-SMN/DTAF ratio was similar in primary neurites at stage 2 (A_1_-A_4_) and in primary (arrowheads) vs. minor neurites (arrows) at stage 2^+^ (B_1_-B_4_). By contrast, a-SMN staining was significantly more intense in axons *vs*. dendrites at stage 3 (C_1_-C_4_: axons 1.79 ± 0.14 and dendrites 1.00 ± 0.09). Data are presented as mean ± SEM of 30 random sampled cells for every stage (2, 2+ and 3) from three different experiments. Statistical analysis was performed by Student’s t-test (** p<0.01). Scale bars: 10 μm.

We next quantified the intensity of a-SMN labeling in primary neurites (stages 2 and 2^+^) and axons (stage 3) *vs*. minor neurites or dendrites respectively (Fig 3). To obtain a reliable quantification, we analyzed rat primary hippocampal neurons co-labeled with #553 rat-specific anti-a-SMN antibody and the non-selective protein labelling dye 5-DTAF, normalizing the #553 fluorescence intensity *vs*. the 5-DTAF intensity. The a-SMN/DTAF fluorescence ratio was similar in all immature neurites of unpolarized stage 2 neurons (Fig 3A_1_–A_4_), and in primary *vs*. minor neurites of stage 2^+^ neurons (Fig 3B_1_–B_4_). By contrast, in polarized stage 3 neurons, a-SMN intensity was significantly higher in the forming axons than in the dendrites (a-SMN relative intensity to green dye 5-DTAF at stage 3 was 1.79 ± 0.14 in axons and 1.00 ± 0.09 in dendrites; Fig 3C_4_,), demonstrating the specific concentration of a-SMN in the rapidly growing axon (Fig 3C_1_–C_4_).

### Efficiency of silencing strategy

**Fig 4:**
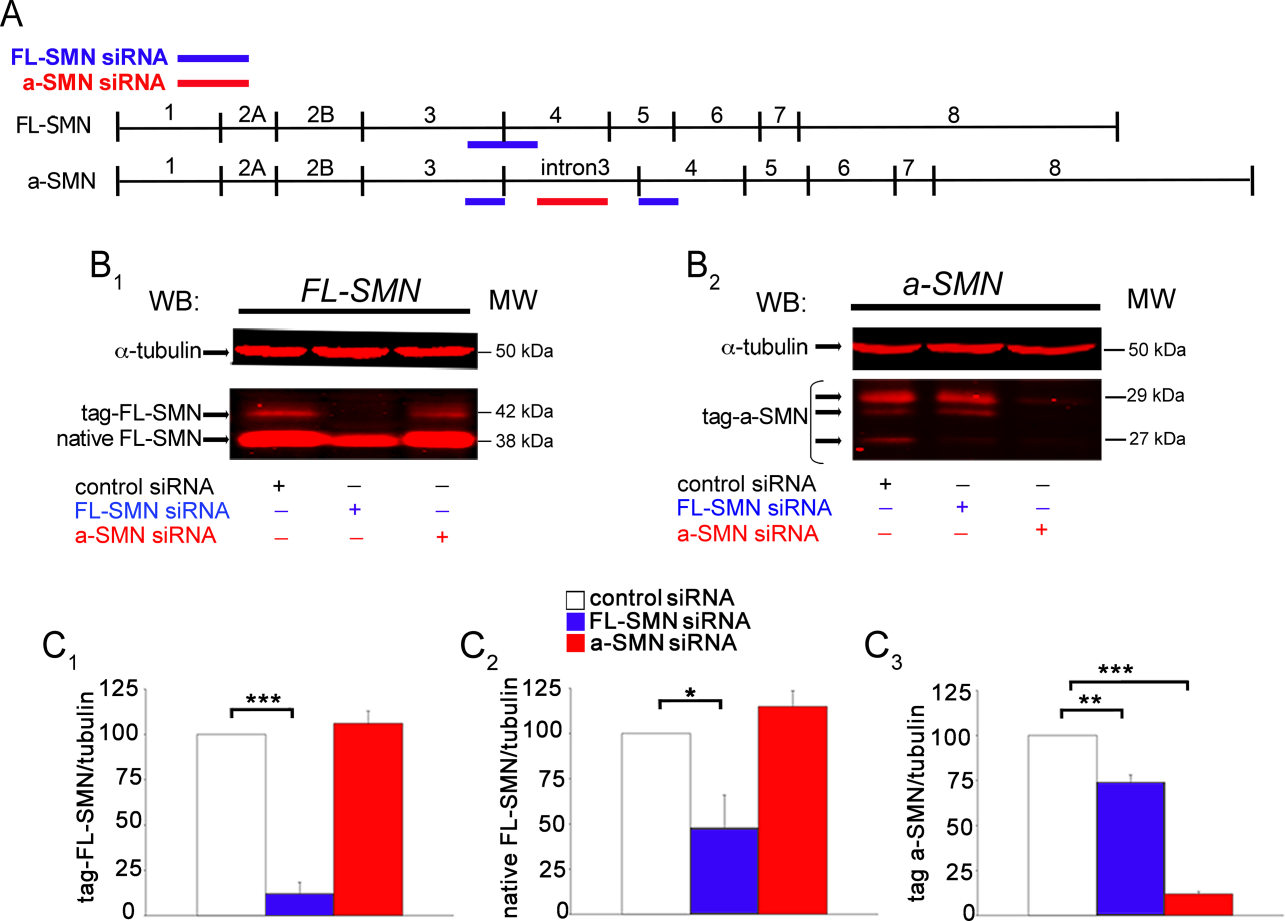
Quantification of FL-SMN and a-SMN silencing in NSC34 motor neurons. (A) Schematic representation of the binding sites of FL-SMN (blue lines) and a-SMN (red line) specific siRNAs along each respective mRNA sequence. Note that the FL-SMN siRNA against the exon 3/exon 4 junction can make a partial annealing to the end of exon 3 and beginning of exon 4 sequence on the a-SMN mRNA (lower blue lines). (B) Western blot analysis of siRNA silencing in NSC34 cells performed with BD Bioscience anti-SMN antibody against the N-terminal region. (B_1_) NSC34 motor neurons were co-transfected with rat FL-SMN tagged construct and control or FL-SMN or a-SMN siRNAs as indicated at the bottom of the blot. Labels on the left indicate the native FL-SMN and the transfected tagged protein as a slightly higher band. Tubulin was reported as loading marker (upper red band). Molecular weights are reported on the right (MW). (B_2_) NSC34 motor neurons were co-transfected with rat a-SMN tagged construct and control or FL-SMN or a-SMN siRNAs as indicated at the bottom of the blot. Transfection of NSC34 with tag-a-SMN led to the expression of three SMN bands, as indicated in the label on the left. Molecular weights are reported on the right (MW). (C) Histograms showing the quantification of immunoreactive ratio of FL-SMN or a-SMN/tubulin in all experimental groups. (C_1_) The 42 KDa tagged-FL-SMN protein expression was significantly down-regulated by FL-SMN siRNA to 12% (***p < 0.001; B_1_, blue bar) compared to control non-target siRNA (white bar). a-SMN siRNA did not modify FL-SMN protein levels (red bar). (C_2_) Native FL-SMN was significantly down-regulated by FL-SMN siRNA to 47% (*p < 0.05; blue bar), compared to control (white bar). No significant difference was obtained with a-SMN siRNA (red bar) vs. control (white bar). (C_3_) The expression of all the transfected tag-a-SMN proteins were down-regulated by a-SMN specific a-SMN siRNA to 12% (***p < 0.001; C_1_, red bar), compared to control non-target siRNA (white bar). Note that the exogenous a-SMN was significantly reduced also by the FL-SMN siRNA (to 74%, **p < 0.01; C_1_, blue bar) probably due to the partial annealing of the FL-SMN siRNA to the exon 3 and 4 sequence of the a-SMN mRNA. Data were normalized versus α-tubulin protein levels and were presented as mean ± SEM of three different experiments. Statistical analysis was performed by Student’s t-test.

To better understand the role of the two SMN isoforms in axons, we next evaluated the differential effect of FL-SMN *vs*. a-SMN silencing on the axonal outgrowth of primary hippocampal neurons. For selective FL-SMN silencing we designed siRNAs directed against the exon 3/exon 4 junction, the only sequence specific for FL-SMN mRNA (Fig 4A, upper blue line). To obtain a-SMN knockdown we designed specific siRNAs targeting the intron 3 region, exclusively retained in the a-SMN mRNA (Fig 4A, lower red line). In all silencing experiments we used the 27-mer Dicer-Substrate siRNAs developed by IDT, more closely mimicking the natural pathway of RNA interference and endowed with higher effectiveness when compared with traditional 21-mer duplexes [54].

Because western blot analysis of primary hippocampal cultures was not sufficiently sensitive for revealing a-SMN expression levels in basal conditions, to quantify the efficiency of our silencing protocol in terms of protein expression we used NSC34 motor neurons co-transfected with either rat FL-SMN or a-SMN tagged constructs, together with FL-SMN- or a-SMN-specific siRNAs (Fig 4). As appropriate controls we also co-transfected NSC34 cells with the same SMN constructs and non-targeting siRNAs.

Western blot analysis revealed that FL-SMN siRNA application was able to down-regulate significantly both the 42 kDa exogenous tagged rat FL-SMN protein (to 12%, p<0.001, blue bar in Fig 4C_1_ and 4B_1_ middle lane upper band) and the 38 kDa endogenous mouse FL-SMN protein (to 47%, p<0.05, blue bar in Fig 4C_2_ and 4B_2_ middle lane lower band), given the very high homology (96.3%) between rat and mouse FL-SMN mRNA. Transfection with tagged a-SMN protein led to expression of three bands of molecular weights ranging between 27 and 29 KDa (Fig 4B_2_), probably due to post-translational processing [32]. a-SMN specific siRNA greatly down-regulated the expression of all three bands of exogenous tagged a-SMN (to 12%, p<0.001, red bar in Fig 4C_3_ and 4B_2_ left lane) without affecting the expression levels of FL-SMN proteins either exogenous or endogenous (red bars in Fig 4B_1_ and 4B_2_, B_1_left lane). By contrast, the application of FL-SMN siRNA was able to reduce significantly also the expression of exogenous a-SMN (to 74%, p<0.01, blue bar in Fig 4C_3_ and 4B_2_ middle lane). This is likely related to the partial recognition by FL-SMN siRNA of the sequences of the exon 3 and/or exon 4 present on a-SMN mRNA (Fig 4A, lower blue lines). Similar results were obtained by probing the blots with the anti-X-press antibody directed against the N-terminal tag of the two transfected SMN isoforms (data not shown).

### FL-SMN *vs*. a-SMN silencing: Effect on axon outgrowth

**Fig 5:**
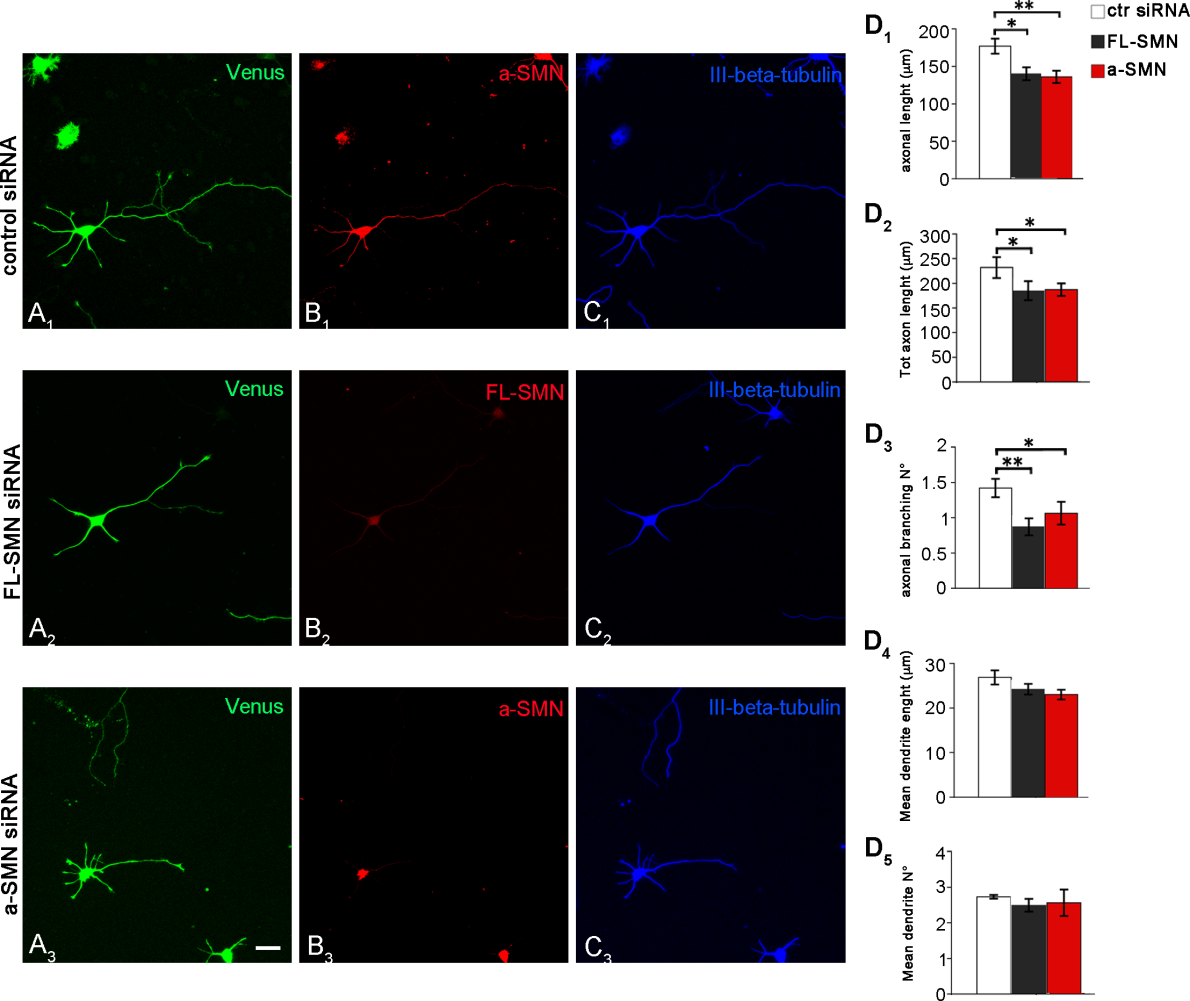
Effect of FL-SMN and a-SMN knockdown on axon growth. Confocal images of stage 3 hippocampal neurons co-transfected with the Venus plasmid (green) and control (A_1_-C_1_) or FL-SMN (A_2_-C_2_) or a-SMN specific siRNAs (A_3_-C_3_), labeled with anti-a-SMN (red, B_1_ and B_3_), anti-SMN (red, B_2_), and III-ß-tubulin (blue, C_1_-C_3_) antibodies. Note that, if compared with neurons treated with control siRNA, both FL-SMN- and a-SMN silenced hippocampal neurons showed after 3 DIV shorter and less extensively branched axons. (D) Morphometric analysis revealed that FL-SMN or a-SMN knock-down were equally effective in reducing axon elongation (FL-SMN and a-SMN *vs*. control siRNA: * p < 0.05, ** p < 0.01: D_1_), total axon length (FL-SMN and a-SMN *vs*. control siRNA: * p < 0.05: D_2_) and axonal branching (FL-SMN and a-SMN *vs*. control siRNA: * p < 0.05, ** p < 0.01: D_3_). By contrast, dendrite length (D_4_) and number (D_5_) were unaffected by either FL-SMN or a-SMN knock-down. Data are presented as mean ± SEM of three independent experiments (axon elongation n > 250 cells, total axon length and axonal branching n > 140 cells, dendrite length and number n > 240 cells). Statistical analysis were performed by means of one-way ANOVA followed by Tukey HSD as post hoc comparison test. Scale bar: 10 μm.

To quantify the effect of SMN isoforms silencing on axon growth, neurons were co-transfected immediately before plating with specific FL-SMN or a-SMN siRNAs and the Venus plasmid expressing a green fluorescent protein, as a transfection marker. Control hippocampal neurons were co-transfected with non-targeting siRNAs and Venus plasmid. Neurons were then plated, fixed 72 hours later and co-stained for SMN-specific antibodies and III-β-tubulin (Fig 5). The majority of neurons were efficiently transfected, as clearly identifiable by the Venus green signal. Morphometric analysis of transfected neurons revealed that both FL-SMN and a-SMN silencing were able to significantly reduce axon outgrowth (compare Fig 5A_1_–5C_1_ *vs*. 5A_2_–5C_2_ and 5A_3_–5C_3_) in terms of axon elongation (i.e., measure of the longest neurite: control siRNA 177 μm ± 10.10, FL-SMN siRNA 140 μm ± 8.70, a-SMN siRNA 136 μm ± 8.1: Fig 5D_1_), total axonal length (i.e., the total length of all axonal branches: control siRNA 232 μm ± 21.18, FL-SMN siRNA 185 μm ± 19.31, a-SMN siRNA 187 μm ± 12.66: Fig 5D_2_) and arborization (i.e., the number of axonal branches: control siRNA 1.42 ± 0.13, FL-SMN siRNA 0.87 ± 0.12, a-SMN siRNA 1.07 ± 0.16: Fig 5D_3_). By contrast, the length and number of dendrites were unaffected by either FL-SMN or a-SMN silencing (dendrite length: control siRNA 26.86 μm ± 1.6, FL-SMN siRNA 24.2 μm ± 1.20, a-SMN siRNA 23.09 μm ± 1.10; dendrite number: control siRNA 2.73 ± 0.05, FL-SMN siRNA 2.49 ± 0.18, a-SMN siRNA 2.58 ± 0.37: Fig 5D_4–5_). The above results suggested that both FL-SMN and a-SMN were participating in axonal growth. No clear difference was evident between the silencing effect of the two isoforms.

### a-SMN and FL-SMN silencing induces “multi-neuritic” neurons display mixed axonal/dendritic phenotype

**Fig 6:**
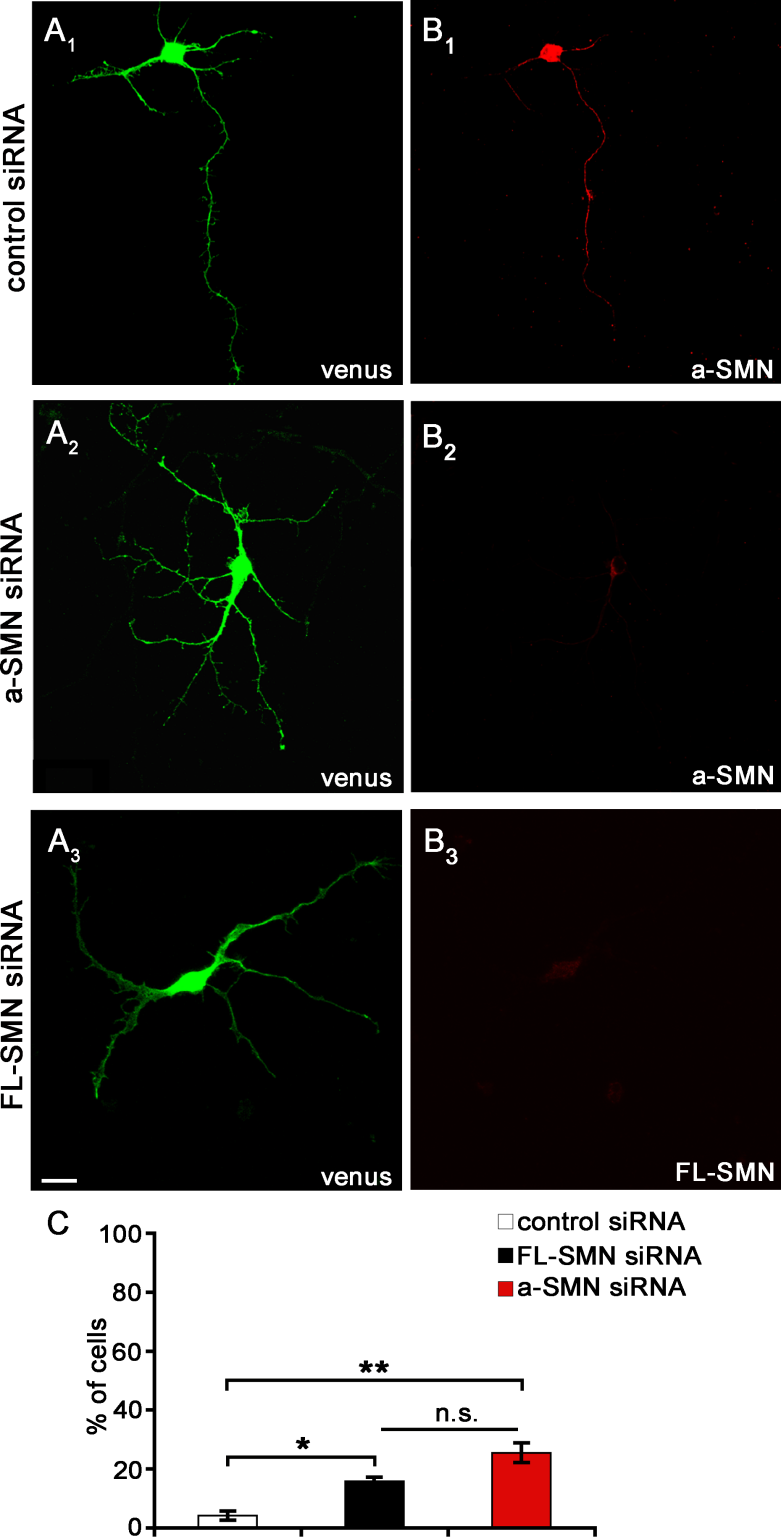
FL-SMN and a-SMN knockdown: Multi-neurite neurons. (A_1_-B_3_) Confocal images of hippocampal neurons co-transfected with control or a-SMN or FL-SMN-specific siRNAs together with Venus plasmid (green, A_1_-A_3_), fixed after 3DIV and labeled with the anti-a-SMN #553 (red, B_1_-B_2_) or anti-FL-SMN antibodies (red, B_3_). After selective a-SMN or FL-SMN silencing, a significant fraction of hippocampal neurons showed several processes with similar length and no clear evidence of axonal polarization. (C) Percentages of Venus^+^ hippocampal neurons displaying multi-neuritc morphology after transfection with control (white bars), FL-SMN SiRNA (grey bars) or a-SMN-specific siRNAs (red bars). Data are presented as mean ± SEM of three different experiments of each group (* p < 0.05; ** p < 0.01; n.s.: not significant). Statistical analysis were performed by means of one-way ANOVA followed by Tukey HSD as post hoc comparison test. Scale bar: 10 μm.

In addition to a reduced axonal growth, the silencing of FL-SMN and a-SMN resulted in the existence of numerous neurons displaying what we called a "multi-neuritic morphology", meaning neurons with multiple long neurites (longer than 40 μm) (Fig 6A_2_–6B_2_ and 6A_3_–6B_3_) rather than the classical phenotype of one long neurite and several short ones (Fig 6A_1_–6B_1_). In particular, morphometric analysis (Fig 6C) revealed that approximately 25% of neurons with reduced a-SMN (25.53% ± 3.40% of Venus^+^ neurons) displayed such abnormality, whereas less than 16% were observed in FL-SMN knockdown (15.91% ± 1.3% of Venus^+^ neurons). By contrast, only a small percentage of the neurons treated with non-targeting siRNA showed a “multi-neuritic” morphology (3,1% ± 1,29% of Venus^+^ neurons).

**Fig 7:**
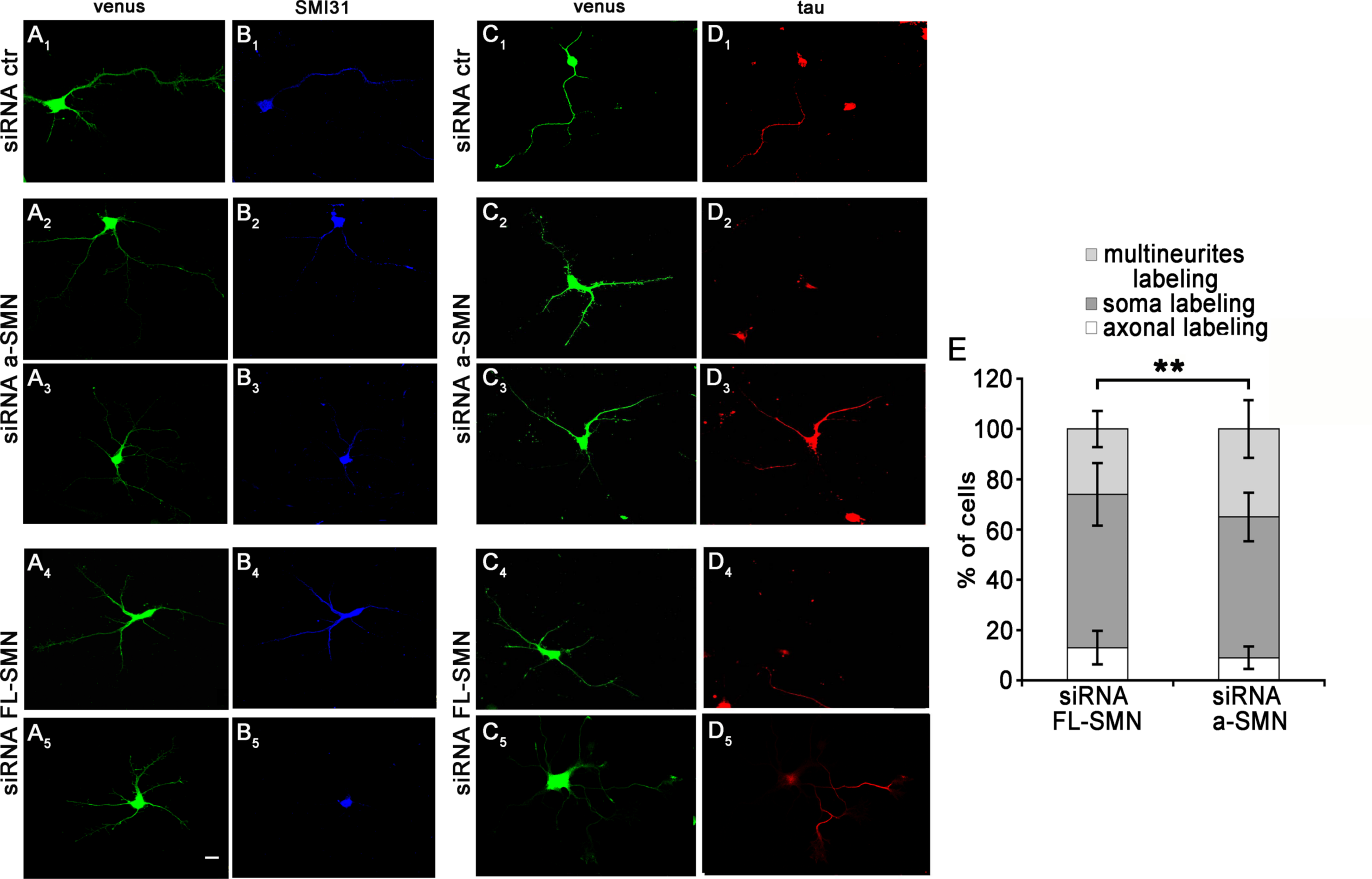
Axon markers SMI31 and tau in multi-neuritic neurons. (A-D) Confocal images of hippocampal neurons co-transfected with control (A_1_-D_1_), a-SMN (A_2_-D_3_) or FL-SMN (A_4_-D_5_) specific siRNAs and the Venus plasmid (green, A_1_-A_5_ and C_1_-C_5_), fixed at 3DIV and labeled with the axonal markers SMI31 (blue, B_1_-B_5_) or tau (red, D_1_-D_5_). While neurons transfected with control siRNA had a single axon labeled by SMI31(A_1_-B_1_), in a-SMN and FL-SMN-silenced neurons displaying multi-neuritic morphology, SMI31or tau axonal staining was mainly in soma (B_5_, D_2_, D_4_) or variably localized in two or more neuritic processes (B_2_-B_4_, D_3_, D_5_). (E) Quantification of axon labeling in individual multi-neuritic neurons. Stacked histograms showing SMI31 distribution in a-SMN and FL-SMN silenced neurons with multi-neuritic morphology. Data are presented as mean ± SEM. At least 70 cells from three different experiments were analyzed. Percent ratio of axonal markers distribution (multineurites/ only soma/axonal labeling) was compared by means of chi-square test (** p<0.01) between the two experimental conditions (a-SMN or FL-SMN siRNA). Scale bar: 10 μm.

To further characterize the phenotype of “multi-neuritic” hippocampal neurons in the a-SMN and FL-SMN loss background, neuronal cultures silenced for FL-SMN or a-SMN were labelled with axonal markers (SMI31 or Tau-1). Confocal analysis of the Venus^+^ transfected neurons revealed that, in contrast to stage 3 control neurons, which displayed selective SMI31 and Tau-1 labelling only in the axon (Fig 7A_1_,7B_1,_ 7C_1_ and 7D_1_), after a-SMN or FL-SMN silencing multi-neuritic cells could display either no SMI31/Tau-1 axonal labelling (i.e., no labelling or only soma labelling) (Fig 7B_5_, 7D_2_ and 7D_4_) or variable axon-specific staining in two or more neuritic processes (Fig 7B_2_–B_4_ and 7D_3_ and 7D_5_). Quantification of the presence of SMI31 or Tau-1 axonal labelling in individual multi-neuritic neurons showed that approximately 56% (± 9.7% of Venus^+^ neurons) of a-SMN silenced neurons did not display any axonal labelling, about 35% (± 11.5% of Venus^+^ neurons) were characterized by axonal labelling in two or more neurites, and very few neurons (9% ± 4.5% of Venus^+^ neurons) showed axonal marker in only one neuritic process (Fig 7E). After FL-SMN silencing, approximately 61% (± 12.4% of Venus^+^ neurons) of silenced neurons did not display any axonal labelling, about 26% (± 7.2% of Venus^+^ neurons) were characterized by axonal labelling in two or more neurites, and only a 13% (± 6.7% of Venus^+^ neurons) of neurons showed a staining in only one neuritic process (Fig 7E).

**Fig 8:**
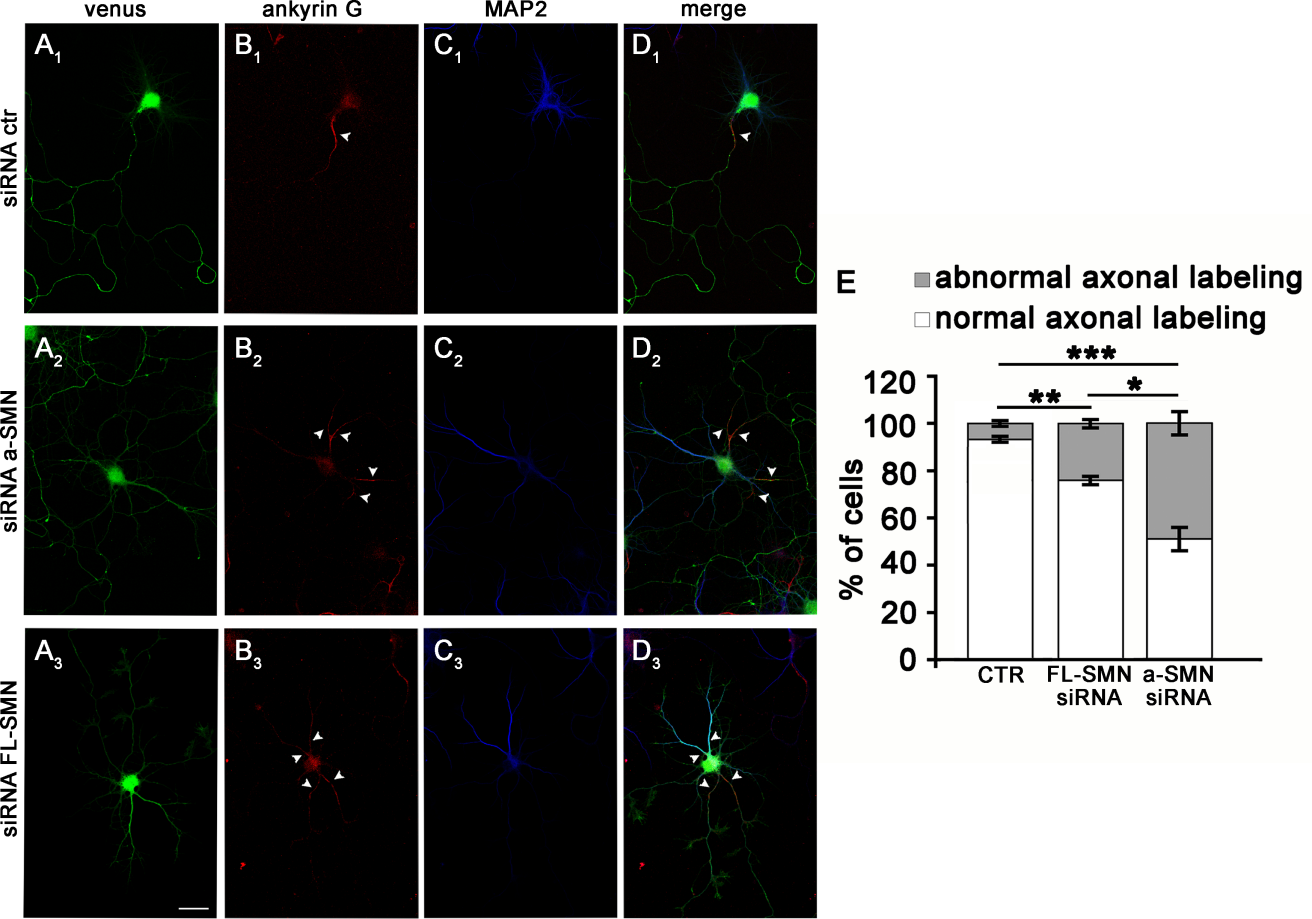
Neuritic processes of multi-neuritic neurons display mixed axonal/dendritic phenotype. (A-C) Confocal images of control (A_1_-C_1_), a-SMN- (A_2_-C_2_) and FL-SMN- (A_3_-C_3_) silenced hippocampal neurons fixed at 8 DIV and co-labeled with the dendritic marker MAP2 (blue, C_1_-C_3_) and the axonal marker ankG (red, B_1_-B_3_), specifically staining the axon initial segment (AIS). (D_1_-D_3_) Merged images. Neurons treated with the control non-targeting siRNA displayed a unique AIS strongly labeled by ankG and different dendrites selectively labeled by MAP2. Staining selectivity was lost in multi-neuritic neurons silenced with a-SMN or FL-SMN, variably displaying axon and dendritic labeling in the same neuritic process. Arrowheads evidence ankG staining. (E) Stacked histograms showing the percent ratio of multi-neuritic neurons with normal vs. abnormal ankG and MAP2 distribution after control, a-SMN or FL-SMN silencing. Data are presented as mean ± SEM of three different experiments. Statistical analysis was performed by chi-square test (** p<0.01, ***p<0.001) among the three experimental groups (control, a-SMN siRNA and FL-SMN siRNA). Scale bar: 25 μm.

The absence of a-SMN or FL-SMN, that in early stages of differentiation results in reduced axonal growth and an increased proportion of aberrantly differentiated cells, may possibly have consequences for the adult neuron phenotype. In fully mature neurons, ankyrin G (ankG) is largely restricted to the axon initial segment (AIS) and reflects the capacity of neurons to maintain neuron polarity and to efficiently transmit action potentials [55]. We therefore analyzed the pattern of distribution of ankG in mature FL-SMN or a-SMN silenced neuronal cultures after 8 DIV (Fig 8). Neurons transfected with Venus and control siRNAs displayed normal ankG^+^ AIS clearly segregated from the MAP2-staining of the dendrites (Fig 8A_1_–8D_1_). In contrast, several neurons co-transfected with Venus and a-SMN or FL-SMN siRNA showed an abnormal ankG labelling, present in multiple neurites including those positive for the dendrite-specific MAP2 protein or in the terminal portion of the axon (Fig 8A_2_–8D_3_). Quantification analysis showed that in a-SMN silenced neurons, ankG was misplaced in 49% ± 4.9% of Venus^+^ neurons; while in the FL-SMN silenced neurons the percentage showing abnormal ankG staining was of 24% ± 1.76% of Venus^+^ neurons.

## Discussion

The involvement of FL-SMN in axonal outgrowth has been postulated in different experimental models in vivo and in vitro [56, 57]. As for a-SMN, it has been reported to be mainly localized to axons and to have neuritogenic effects in NSC34 cells [32, 58]. To better understand the specific role of the two isoforms, we utilized hippocampal neuronal cultures as a reproducible and well characterized tool, especially suitable to study processes of neuronal polarization and axonal growth, maintenance and functionality [46]. The present experiments provide two sets of data. First, by analyzing its subcellular localization in primary cultures of hippocampal neurons, we confirm here that a-SMN is preferentially distributed in the axonal compartment. Second, by specifically silencing FL-SMN or a-SMN proteins, we show that both proteins play a role in axon growth and neuronal polarization, as their selective down-regulation reduces axon length without affecting dendritic arborization. Furthermore, a-SMN and FL-SMN silencing is capable of inducing the growth of multi-neuritic neurons, i.e., neurons impaired in the differentiation process of selecting a single axon out of multiple neurites.

### a-SMN is enriched in the axon of differentiated hippocampal neurons

FL-SMN is expressed ubiquitously and is present in cytoplasm and nucleus of every cells, with main functions in the biogenesis of spliceosomes and the maturation of pre-mRNAs [33–36]. It is also localized in dendrites and at axonal branch points of cultured primary motor neurons [59], in growth cone and in neurites of differentiated P19 cells [6], in neurites and growth cones of hippocampal neurons [43]. a-SMN protein is found in neuronal and non-neuronal tissues, albeit at different levels, and clearly shows developmental down-regulation. In spinal cord, a-SMN is mainly expressed in motor neurons, and particularly localized in axons [32]. When expressed in mouse motor neuron-like NSC-34 cells, a-SMN localizes to soma and neurites and is excluded from the nucleus [58]. Here we analyzed the distribution of endogenous a-SMN in the different developmental stages of cultured hippocampal neurons and demonstrated that, while it is widely distributed in all neurites of unpolarized neurons until stage 2/2^+^, as neuronal polarization progresses it becomes specifically enriched in the growing axon of stage 3 and stage 4 neurons. Unlike FL-SMN [6, 43], a-SMN appears to be absent from the growth cone. The specific localization of a-SMN in the axonal compartment is consistent with its neuritogenic effect in NCS-34 cells. [32, 58].

### FL-SMN *vs*. a-SMN function in axonal growth

Motor neurons from severe SMA transgenic mice manifested reduced axon elongation [59, 60], and specific FL-SMN silencing was related to evident reduction of neurite production and length of NSC-34 cells [61], to neurite degeneration of cultured mouse motor neurons [62] or reduced neurite outgrowth in motor neurons differentiated from embryonic stem cells [63]. In keeping with those previously published data, we confirmed here that FL-SMN protein silencing to approximately 15% determined a significant reduction in the elongation, total length and arborization of the growing axons without affecting dendrites, supporting the notion that FL-SMN loss of function in neurons is to a great extent related to impairment of the normal process of axon growth and stability. Silencing of a-SMN determined as well a reduction of axon length and branching with no alteration of dendritic elongation and morphology, confirming a specific role of a-SMN in axon outgrowth [45, 32]. The effect of FL-SMN on axonal growth has been ascribed to its numerous interactions with ribonucleoprotein granules and proteins involved in actin dynamics, mRNA transport, local translation [57, 64]. For some of these proteins, the mechanism of interaction is still not clear, like for plastin3 that in zebrafish rescues axon defects due to SMN deficit through calcium regulation and actin bundling [65]. For others, a direct binding was described to specific sites on FL-SMN sequence. Heterogeneous nuclear ribonucleoprotein R (hnRNP R) is required to bind to FL-SMN to promote axon growth by mean of translocation of beta-actin mRNA to growth cones [44]; likewise, cGP15/neuritin mRNA bound to ELAV-like RNA binding protein HuD/SMN complex rescues motor axon defects caused by a reduction of SMN in zebrafish [41]. Both hnRNP and HuD need to be bound to SMN for axonal localization [44, 41]. The binding site is localized in the Tudor domain [40] which is involved in interactions with several proteins carrying RGG/RG motifs [64] and is, together with the C-terminal YG-box, a mutational hot spot in SMA [66]. SMA patient-derived missense mutations in the Tudor domain significantly impaired HuD-SMN association [40]. Notably, when SMA patient-derived missense mutations or small intragenic re-arrangements located in the Tudor domain were introduced in the a-SMN sequence, axon elongation induced by a-SMN expression was consistently altered [45]. The Tudor domain is located in the N-terminal part of FL-SMN that is identical in a-SMN [32] and may be similarly important for a-SMN effect on axon growth, as a site for anchoring a-SMN to other proteins or mRNA involved in neuronal development, growth and transport (such as hnRNPs, HuD, neuritin). On the other hand, the C-terminal domain is equally if not more important for axon growth in both proteins. In fact, mutations in the C-terminal part of a-SMN abrogated completely the axonogenic effect in NSC-34 [45], while selective overexpression of the FL-SMN C-terminal domain promoted neurite outgrowth similar to full-length protein and could rescue the SMN knock-down effects in PC12 cells [67]. Different domains in the C-terminal sequence of FL-SMN are important for the axonal role of the protein: the QNQKE motif within exon 7 is required for axonal localization of FL-SMN [68, 69], while the proline-rich domain binds Profilin 2 and regulates its phosphorylation state and F-/G-actin ratio, promoting neurite outgrowth [70]. The in-frame stop codon present in the intron 3 sequence causes a-SMN to have a different C-terminal tail that lacks these domains [32], and the mechanisms by which a-SMN C-terminus defects abrogate axonogenesis are yet to be clarified.

### FL-SMN *vs*. a-SMN function in axonal polarization

Besides the negative effect on axonal length, our data revealed that a-SMN and FL-SMN silencing were capable of impairing the program of axon development. In fact, a consistent fraction of hippocampal neurons silenced for a-SMN or for FL-SMN failed to develop a prevailing axon (what we called “multi-neuritic” neurons). Axonal markers Tau and SMI31 were largely not expressed or unselectively localized in more than one neurite, as if these neurons were unable to proceed with the normal polarization that is typical of neuronal cells. Moreover, the AIS specific protein ankG was mislocated in multiple neurites, including those positive for the dendrite-specific MAP2 protein, or in the distal neurite. To our knowledge, this is the first time that down-regulation of SMN proteins is reported to cause loss of specific ankG localization and axonal specification. The segregation of specific proteins during neuronal polarization depends on the physical assembly and stability of the axon initial segment (AIS), a specialized compartment that separates the axonal and somatodendritic components and integrates presynaptic inputs to generate action potentials [71, 55]. The key protein for AIS development and stability is ankG, a scaffold protein specifically located in the AIS in the proximal segment of the axon [72, 73] where it interacts with all the submembrane and cytoplasmic layers of the AIS through its numerous binding domains and thus coordinates the localization of all AIS known components [55]. AnkG is indispensable for the localization and retention of other AIS proteins, for the integrity of AIS and the preservation of axonal polarity. Transgenic mice lacking ankG also lack an AIS [74], and cultured neurons where ankG has been knocked-down never develop an AIS [49]. In mature hippocampal cultures, silencing ankG causes the axon to acquire molecular and structural properties of a dendrite, i.e., MAP2 staining and dendritic spines formation [75]. The presence of MAP2 in association with ankG in the same neurite observed in a-SMN and FL-SMN silenced neurons, together with the mislocalization of axonal markers, is thus a clear indication of failure of axonal specification. The formation of axon-dendrite polarity is regulated by molecules affecting actin dynamics [76] or microtubule dynamics [77, 78, 79]. The mechanisms responsible of recruiting ankG to the proximal axon to initiate the organization of AIS are still an unresolved question, but it has been shown that AIS formation is also dependent on cytoskeleton dynamics, requiring cooperative interactions of ankG with the microtubule regulating end-proteins EBs in the proximal axon [80] and a cytoskeletal structure of αII-spectrin, βII-spectrin and ankB in the distal axon [81]. Another important mechanism for the control of AIS formation is phosphorylation of ankG and its binding partners [82, 83]. Further investigations are needed to clarify the role of FL-SMN and a-SMN in these processes. As abnormalities in the microtubule polymerization and organization has been described in SMA mice models [84, 85] and given the role of FL-SMN in signaling pathways regulating the actin cytoskeleton, one might speculate an effect on the organization of neuronal cytoskeleton. In cultured neurons, microtubule-stabilizing drug taxol or Glycogen synthase kinase-3 beta (GSK-3beta) inhibition induced multiple axons that are positive for ankG and Tau [86, 87] and have a functional AIS [88]. On the contrary, in hippocampal cultures transfected with a constitutively active GSK-3beta, 49% of neurons showed a phenotype similar to what we observed after a-SMN or FL-SMN silencing: no axon, neurites significantly longer than normal dendrites and an abnormal localization of dendritic marker MAP2 and axonal markers Tau-1, GAP43 and Synapsin I in all neurites [87]. GSK-3beta is a multifunctional serine/threonine kinase known to be important in many biological processes ranging from Wnt signaling pathway to MT dynamics to astrocyte migration [89, 90]. Its role in determining neuronal polarity is regulated by Akt/PTEN signaling [87]. Interestingly, PTEN depletion was shown to rescue axonal growth defects of SMN deficient motor neurons and to decrease disease severity in a SMA mouse model [91, 92]. Moreover, GSK-3 chemical inhibitors have been shown to increase SMN levels in motor neurons [93], an inhibitor of GSK-3beta was found to extend the median survival period of transgenic SMA delta7 mice [94], and the neuroprotective effects of butyrate-based compounds on SMA delta7 mice were demonstrated to be exerted through modulation of Akt/GSK-3beta pathway [95].

a-SMN siRNA caused multi-neuritic morphology and misplacement of ankG in a percentage of neurons significantly higher compared to FL-SMN siRNA. However, our data did not evidence a clear difference between the effect of silencing FL-SMN or a-SMN. As the two isoforms share the same N-terminal portion [32], a partial overlapping in their actions is possible. Some redundancy between FL-SMN and a-SMN would not be surprising, especially in the nervous system, given the vital role of the proteins in numerous cellular processes [64]. Furthermore, due to the high degree of similarity between the FL-SMN and a-SMN mRNA sequences, we were unable to reduce FL-SMN expression without decreasing also a-SMN. We therefore cannot exclude that the effects observed after FL-SMN silencing might be partially due to a decrease of a-SMN. On the other hand, the reason why, after silencing a-SMN or FL-SMN, some neurons showed an impairment of axon growth while others failed to even develop an axon may depend on the heterogeneity of the transfection efficiency causing different degrees of protein expression. Likewise, SMA patients present a large spectrum of phenotype severity determined by SMN2 copy number [96].

Defects in axon growth and arborization has been described in numerous in-vivo and in vitro SMA models [57]. Here we show that loss of FL-SMN or a-SMN results in multi-neuritic neurons with missing or misplaced Tau and SMI31 staining and loss of AIS, suggesting that the role of these proteins in neuronal development is more complex than the sole axon elongation. Neurons are highly specialized cells of the nervous system that receive, process and transmit electrical signals critical for normal living functions. The organization in axonal and dendritic compartments is fundamental for neuronal signal transmission and AIS is indispensable to maintain neuronal polarization and to initiate action potentials. Mislocation of axonal markers is clearly correlated with neuronal dysfunction: for example, loss of the polarized distribution of Tau is a key early deficit in neurodegenerative diseases [97], while SMI31 decrease was related to the degree of neuronal impairment [98]. AIS integrity is related to the health of the nervous system, as ankG deficiency contributes to the pathology of several neurological diseases (e.g., epilepsy, schizophrenia, bipolar disorder, autism spectrum disorder, and Alzheimer’s disease) [99].

## Conclusions

SMA is characterized by spinal motor neuron degeneration, leading to progressive denervation of skeletal and intercostal muscles, muscle weakness, paralysis, and eventual death due to respiratory failure [100]. Evidences from the large number of invertebrate animal and mouse models generated to study the pathomechanisms of this devastating disease suggest that axonal degeneration or otherwise compromised function of motor neurons is an early event in SMA, leading to the concept of SMA as an axonopathy [57]. Our data are consistent with this concept, evidencing how the role of a-SMN and FL-SMN in the neuronal development is not limited to a diminished axonal elongation but involve the regulation of neuronal polarization and the correct organization of axonal and dendritic compartments, that are vital for neuronal function and survival.

## Supporting information

S1 Fig #553 anti-rat-a-SMN antibody specificity. Odyssey Infrared Imaging System (LI-COR) acquisition of western blot analysis on rat embryo cortex and hippocampus lysates with #553 anti-rat-a-SMN antibody (green) and BD Bioscience SMN antibody (red). Note the specific BD anti-SMN immunoreactive band corresponding to FL-SMN (migrating at about 37 kDa, lower red band) and the specific a-SMN band recognized by the #553 antibody (green band), with a molecular weight of approximately 29KDa. Actin was also reported as loading (upper red band, ~ 40 kDa). Molecular weight (MW) markers are shown on the left. Antibodies are indicated in grey on far left.
(TIF)
