# Supplementary figures and images for: Axon outgrowth and neuronal differentiation defects after a-SMN and FL-SMN silencing in primary hippocampal cultures
Source: PLoS One. 2018 Jun 14;13(6):e0199105. doi: 10.1371/journal.pone.0199105 (PMC6001960; doi:10.1371/journal.pone.0199105)

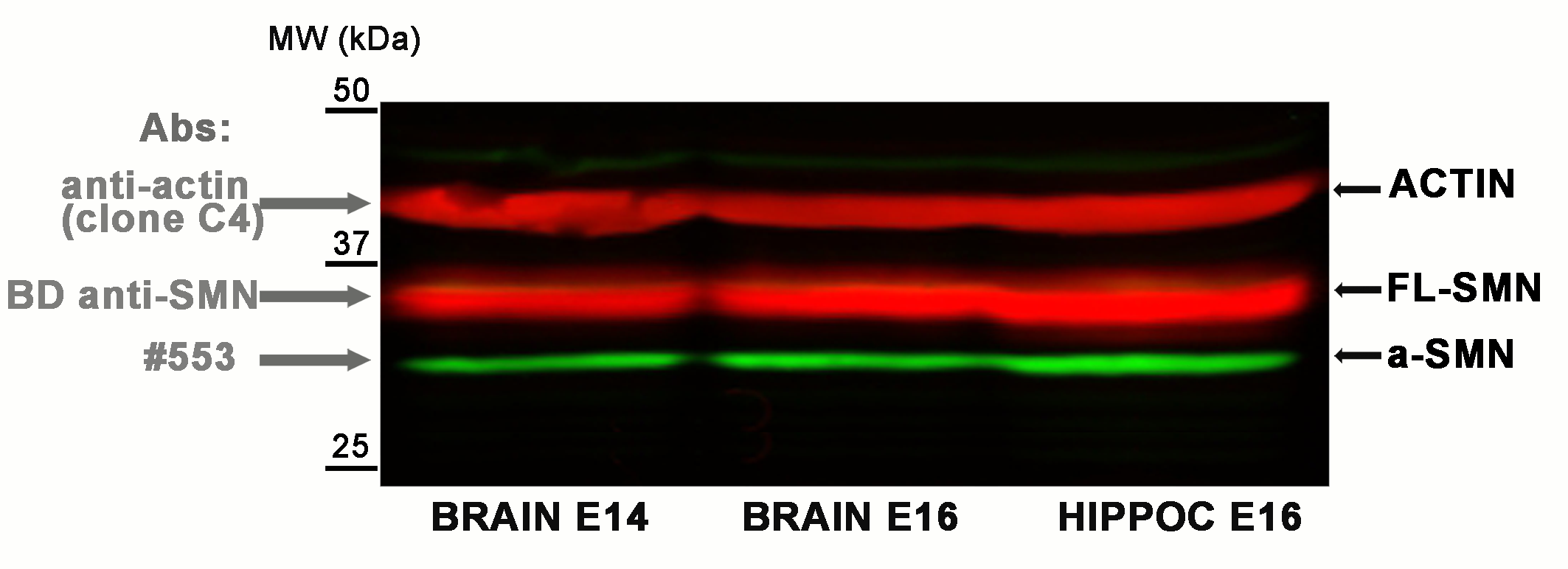

Supplement: S1 Fig — Odyssey Infrared Imaging System (LI-COR) acquisition of western blot analysis on rat embryo cortex and hippocampus lysates with #553 anti-rat-a-SMN antibody (green) and BD Bioscience SMN antibody (red). Note the specific BD anti-SMN immunoreactive band corresponding to FL-SMN (migrating at about 37 kDa, lower red band) and the specific a-SMN band recognized by the #553 antibody (green band), with a molecular weight of approximately 29KDa. Actin was also reported as loading (upper red band, ~ 40 kDa). Molecular weight (MW) markers are shown on the left. Antibodies are indicated in grey on far left. (TIF) [file pone.0199105.s001.tif]
